# Event and Comment

**Published:** 1905-02-15

**Authors:** 


THE
### DENTAL REGISTER.

Vol. LIX. February 15, 1905. No. 2.

### EVENT AND COMMENT.

### The McKellops' Library.

It is reported that Washington University of St. Louis has purchased for its dental department the dental library collected by the late Dr. H. J. McKellops. If this is true and the library has been kept intact since his death, the dental department of Washington University is to be congratulated for doing a very wise thing. At the time of Dr. McKellops' death this was probably one of the most complete libraries in the world of dentistry. Too much can not be said in favor of preserving our literature. It is not only the only means of recording our development, but it can be utilized in educational work to develop a taste for literature and to stimulate research into all lines of professional learning. There is no question but all the classics of our literature will be preserved by appreciative practitioners, but much that now seem of little importance will some day be useful links in completing historical data. It would seem that our dental colleges are specially good places for the accumulation of these libraries, but with three or four exceptions the colleges make no persistent effort to gather and preserve even the text books of the time. The effort of Dr. Arthur C. Black, of Northwestern University, to establish a dental periodical clearing-house is a good move to encourage. Every dentist should send him any old books he may have that he is not using.

**Reciprocal Professional Relations.**

A great deal is being said and written in our journals about transfer of licensed practitioners from one State to another on some proper basis of reciprocity. The States of New York, New Jersey and Pennsylvania have agreed to a plan of transfer that may help to bring about a solution of this problem. One of the widely-circulated journals printed in its December issue a communication from a licensed practitioner of New York, who complained of the unfair treatment he received from the Colorado Board of Examiners, which has been answered in a communication from a member of the Colorado Board, and is published in the February issue of the same journal. The charge is weak enough on its face to justify the Colorado Board for its action. But the reply makes it very clear that the Board could not have acted otherwise and keep faith with its duty to the people. In this connection it also develops that the communication in the first place was intentionally fictitious in large part, and the editor acknowledges that he thought as much when he accepted it for publication; but justifies his action by the fact that the article was well written and attractive from a literary standpoint, and that he hoped it would be the means of opening up a discussion of the subject of State reciprocity.

It has always been, so far at least as we have known it, the idea that our journal literature was based on facts, tentative at least, if not always scientifically determined. It comes as a new idea, that fiction also is to have an acknowledged place in our periodical literature. Poetry or rhyme, at least, has been used in our scientific discussions when it served as an illustration or to make a fact more impressive in its statement; but pure or even adulterated and masked fiction ought to have no more place in a scientific journal than in the Bible, even though it be used in discussing a matter of business relationships. As for the matter of promoting discussion by such means we have our own opinion, but we are perfectly willing that other editors shall use their space for this purpose should they so desire. There can be but one mind as to the desirability of an equitable plan for transferring legalized practitioners from one State to another, and the plan should be discussed freely in order that the most equitable arrangement shall be adopted; but no possible good can come from fictitious or unfair incriminations which are more likely to hinder than help on the cause.

**A Case of Identification.**

Several journals have printed a marked chart showing the dental work done in the mouth of a young woman found dead on Cutler Mountain December 17th. It is hoped by the publication of this chart that some dentist will be able to identify the work and help to solve this mysterious murder. The most characteristic marks are two gold bridges in the upper jaw. The right one includes the cuspid, two dummy bicuspids and the first molar as an abutment. The left one includes the first bicuspid abutment to second molar abutment, the second bicuspid and first molar being dummies, the molar of this bridge is made of a bicuspid dummy. It is possible that some dentist might be able to identify this work should it happen to fall under his notice; but such bridges are so frequently made that the chances of identification are very slight. Any dentist who has lost such a patient would do well to investigate this case. For further information apply to Chief of Police, Colorado Springs, Colorado.

**Elimination of General Science Studies from the Dental Curriculum.**

The president of the Institute of Dental Pedagogics, in his annual address, recommended, as the dental curriculum will be much crowded because of the return to a three-year course, that the purely fundamental science subjects to be left to the preparatory schools. We presume he meant such subjects as chemistry and biology. The editor of the *American Journal,* commenting on the matter, seems to favor the teaching of these subjects even more thoroughly in our colleges, because they should be restated, not as isolated facts, but in their relations to the science and art of dentistry. Both are steps in the right direction. When we reach the point where a full scientific course in a good high school or academy is made the basis of admission to the dental course, then we can drop the teaching of such fundamental sciences as are well taught in the preparatory schools and these subjects can be taught by educated dental science teachers as applied sciences. It is a great burden put upon the colleges to spend so much time in endeavoring to teach subjects which can just as well be taken in the preparatory schools, and should be required before admission to the course. Something of this kind must be done or the technical side of education will retrograde, because what may be called the art sciences of medicine, which have such important relations to clinical dentistry are rapidly extending until it requires each year more and more time for their presentation. Then the purely technical subjects are also continually growing until it seems that it will be necessary, unless more time is taken in which to impart all the desirable instruction, that many of the subjects we have heretofore considered important should be cut out of the curriculum.

**Professional Emancipation.**

The editor of *Dental Summary,* contrary to his usual habit, in a six-page editorial, criticises in a somewhat sarcastic vein, a paper read before the Indiana State Dental Society, which he publishes with a full discussion in the February issue of the *Summary.* The paper urges the profession to prevent in some way the dental dealers from "exploiting dental students" at the beginning of practice by selling them on credit goods which they do not need, thus burdening them with debt and putting them under obligations to buy all their supplies from the house which starts them in business, The paper also proposes that no exhibit of supply houses shall be permitted at any meeting; that the profession should publish its own journal independent of any supply house; and lastly that a systematic movement be started to foster original research in dental science and art. The paper puts these ideas, which are not new, in rather offensive terms, and brother Bethell sets the matter out in a truer light. We publish in another place two extracts from the discussion which also consider the matter in a saner manner. So far as the main points in the controversy are concerned, there is, in a word, no good reason for any agitation of these subjects at this time. The fact is that it is the "house organs" that are preserving and recording the literature of the profession. The only independent organ the profession has is not self-supporting, because the profession will not give it that financial support that is necessary to carry it on without the advertising it gets from supply houses. The essayist claims that the medical profession supports several independent organs. This is not true, as every medical journal published has a large advertising patronage, and not one, not even the journal of the American Medical Association, could live without this patronage. We read all the dental journals published independently and by supply houses, and we are sure that we can consistently affirm that the reading pages of all our journals are practically free from advertising matter, which can not be said of the medical journals, as a class. There are a few medical journals which admit no advertising to the reading pages, but a great many of them are not over professional in this matter. We are pleased to say that in the four years we have edited this "house organ" our publishers have made no demand on us for our reading space. In fact, when we assumed the editorial function the only stipulation made as to this matter, was that we should have full charge of the reading pages and print whatever we saw fit, except that we should not admit matter to our pages which belonged in the advertising pages, and that we should not maliciously slander anybody and get the publishers into a lawsuit. We have on both sides religiously kept this agreement, and we see no evidence of a different condition existing in other "house organs." The other matters which the essayist wants regulated we are willing to leave entirely to the dental colleges, the dealers and new graduates for adjustment, as they are personal and private matters, and we can in no way see that they materially threaten the dignity of the profession. We have many times in our pages commended the dealers' exhibits as educational factors in our society meetings. They should be encouraged to assist in making our programs attractive, but not to the extent of overpowering the more essential features. We need original research and lots of it. We have had but little of it, except what has been done by college men, and it will always be to these men we shall have to look for this kind of work, It will come in due time, in fact much of this kind of work is now being done, but it is slow and tedious work and results can not be looked for as frequently as we should like. It may be that some rich man will some day endow a dental research laboratory, or it may be possible that the Carnegie Institute may take on this work. We do not know what is to become of the Evans' millions which were left to found a dental museum in Philadelphia. It may be that this will be our hope for the future of our sciences.

**Our New Cover.**

Our printers surprised us with a new cover last month. We always liked our green one, as it was "fresh." We suppose our printer took his cue from our editorial in the January issue, in which we took occasion to brag about our long life; he probably thought the golden yellow more in keeping with our years; as Shakespeare puts it, "Our way of life is fallen into the sere, the yellow leaf." We shan't quarrel with our circumstances, but try to get used to it.

**State Dental Laws.**

The following States require all applicants for registration shall be graduates of recognized schools and also pass an examination before the Board: Colorado, Iowa, Kentucky, Louisiana, Maryland, New Jersey, New York, Ohio, Oregon, Pennsylvania, Vermont, Washington, Wisconsin.

The following States require all applicants to take an examination, but they need not necessarily be graduates: Alabama, Arizona, Arkansas, California, Connecticut, District of Columbia, Idaho, Indiana, Maine, Massachusetts, Michigan, Mississippi, Missouri, Montana, New Hampshire, North Carolina, South Carolina, North Dakota, South Dakota, Rhode Island, Utah, Virginia, West Virginia.

The following States register without examination graduates of reputable colleges: Kansas, Nebraska, Nevada, Oklahoma, Tennessee, Illinois, Texas.

The following States exchange licenses with New Jersey under the Asheville resolution: District of Columbia, Indiana, Michigan, Tennessee and Utah; Pennsylvania and New York by special ageement.—We extract these statistics from the legislative department of the February *Items of Interest*.

